# Therapeutical Notes

**Published:** 1900-06-23

**Authors:** 


### THERAPEUTICAL NOTES.

### Alopecia.

G. Macgowan advocates the use of trikresol in this disease as a local application, either pure or in the form of an ointment. The drug is rubbed into the scalp every fifth day, and the average time required to effect a cure is about 2½ months. Trikresol is less irritating and painful than carbolic acid, and the results obtained by it are stated to be satisfactory.—*Amer. Jour. Med. Sci.*, January.

### Pertussis.

The following formulas may be used instead of the common belladonna mixtures:—

(1) Resorcin, gr. xxx.

Antipyrin, gr. xv.

T. belladonnæ, viii.

T. opii, ♏iii.

Syr. codeinæ, ʒijss.

Syr. tolut., ʒv.

Aq. destill., ℥ijss.

M. S. ʒi. every six hours.

(2) Bromoform, ♏x.

Alcohol, ♏xlv.

Aq. carui, ad. ℥i.

M.

S. ʒii. every two hours.

(3) Zinci sulph., gr. iv.

Liq. atropiæ, ♏xii.

Syr. zingib., ʒiv.

Aq. cinnam., ad. ℥iij.

M.

S. ʒi. two or three times a day. The zinc and atropine may be gradually increased.

—*Merck's Archives*, December.

### Tinnitus Auruin.

Robin and Mendel speak well of the fluid extract of cimicifuga racemosa in daily doses of about 30 drops. They consider that it has a sedative effect on the aural circulation as well as upon the auditory nerve, and find it an excellent remedy for certain forms of tinnitus.

